# Isolation of novel simian adenoviruses from macaques for development of a vector for human gene therapy and vaccines

**DOI:** 10.1128/jvi.01014-23

**Published:** 2023-09-15

**Authors:** Wendong Lan, Lulu Quan, Yiqiang Li, Junxian Ou, Biyan Duan, Ting Mei, Xiao Tan, Weiwei Chen, Liqiang Feng, Chengsong Wan, Wei Zhao, James Chodosh, Donald Seto, Qiwei Zhang

**Affiliations:** 1 BSL-3 Laboratory (Guangdong), Guangdong Provincial Key Laboratory of Tropical Disease Research, School of Public Health, Southern Medical University, Guangzhou, Guangdong, China; 2 Institute of Medical Microbiology, Jinan University, Guangzhou, Guangdong, China; 3 The Fifth Medical Center, Chinese PLA General Hospital, Beijing, China; 4 State Key Laboratory of Respiratory Disease, Guangzhou Institutes of Biomedicine and Health, Chinese Academy of Sciences, Guangzhou, Guangdong, China; 5 Department of Ophthalmology and Visual Sciences, University of New Mexico School of Medicine, Albuquerque, New Mexico, USA; 6 Bioinformatics and Computational Biology Program, School of Systems Biology, George Mason University, Manassas, Virginia, USA; 7 Key Laboratory of Viral Pathogenesis & Infection Prevention and Control (Jinan University), Ministry of Education, Guangzhou, Guangdong, China; International Centre for Genetic Engineering and Biotechnology, Trieste, Italy

**Keywords:** simian adenovirus, adenovirus vector, seroprevalence, replication-deficient, Gibson assembly, E1 region, E3 region, E4orf6, vaccine vector, gene therapy vector

## Abstract

Both human and non-human simian adenoviruses (HAdVs and SAdVs, respectively) have been used as gene therapy and vaccine vectors. The high prevalence of HAdVs and the neutralizing antibodies associated with prior infection, may limit HAdV-based vector use in human subjects. To overcome this drawback, a vector derived from a newly isolated and characterized macaque adenovirus was constructed. SAdVs (33.9%) were screened from 115 SAdV fecal samples collected at a zoological park. One novel SAdV was isolated and the whole genome was sequenced and analyzed. The pre-existing neutralizing antibody levels were very low against this isolate (10%). Interestingly, SAdV vector constructs that lack E3 region could not produce infectious progeny in HEK293 cells, suggesting that the E3 region is necessary for SAdV replication. The absence of E3 region could be compensated for by replacement with HAdV-5 E4orf6; the resultant construct could replicate well in HEK293 cells. The enhanced Green Fluorescent Protein (eGFP) was inserted into SAdV E3 region and expressed at high level. One-step growth curve showed that the replication of the SAdVs with HAdV-5 E4orf6 substitution and E1/E3 deletion was similar to that of wild-type SAdVs in HEK293 cells, but the modified SAdVs were replication-deficient in A549 cells which lack HAdV-5 E1A and E1B. Finally, we demonstrated that GZ3-12 could infect cells expressing hCAR or hDSG2 receptors. The successful isolation, characterization, and modification of novel SAdVs provide a potentially important vaccine and gene therapy candidate and a new strategy for the rapid acquisition and development of non-HAdV-based alternative vectors for human health applications.

**IMPORTANCE:**

Adenoviruses are widely used in gene therapy and vaccine delivery. Due to the high prevalence of human adenoviruses (HAdVs), the pre-existing immunity against HAdVs in humans is common, which limits the wide and repetitive use of HAdV vectors. In contrast, the pre-existing immunity against simian adenoviruses (SAdVs) is low in humans. Therefore, we performed epidemiological investigations of SAdVs in simians and found that the SAdV prevalence was as high as 33.9%. The whole-genome sequencing and sequence analysis showed SAdV diversity and possible cross species transmission. One isolate with low level of pre-existing neutralizing antibodies in humans was used to construct replication-deficient SAdV vectors with E4orf6 substitution and E1/E3 deletion. Interestingly, we found that the E3 region plays a critical role in its replication in human cells, but the absence of this region could be compensated for by the E4orf6 from HAdV-5 and the E1 expression intrinsic to HEK293 cells.

## INTRODUCTION

Adenoviruses are non-enveloped, icosahedral, double-stranded DNA viruses that infect a broad range of vertebrates (1). Paradoxically, adenoviruses are not only pathogenic microbes, but are also versatile platforms for the development of therapeutic agents for humans. Within the *Adenoviridae* family, the mastadenovirus genus includes human adenoviruses (HAdVs) and non-human simian adenovirus (SAdVs) (2, 3), comprising a common lineage (2). HAdVs are long-studied human pathogens (4, 5), transmitted through fecal-oral and/or respiratory/ocular routes and are associated with a wide spectrum of human diseases including hepatitis, gastroenteritis, keratoconjunctivitis, and acute respiratory disorders (6 – 10). HAdVs have been intensively studied as pathogens, and have also served as model organisms for revealing immunological (antigen presentation to T-cells), cellular (eukaryotic DNA replication), molecular (mRNA splicing), and biological mechanisms (11 – 13). HAdVs are foundational agents for human gene therapy and vaccine vectors despite their prominence as human pathogens (14 – 16).

Due to the high transmissibility of HAdVs, the prevalence of anti-HAdV neutralizing antibodies (nAb) are very high in adult human populations, especially for HAdV-5, which is most widely used as HAdV vector. For example, surveys showed seropositivity rates of 58.4%, 63.8%, and 35.3% for HAdV-4, HAdV-7, and HAdV-26 in China, respectively (17, 18). However, prior studies have reported a 30% nAb prevalence to HAdV-5 in UK (19), 35–70% in North America (20, 21), 70–100% in Asia (22, 23), 60–100% in Africa (24, 25), and 70% in Brazil (26). Due to these presumably widespread and pre-existing nAb levels, and the robust host immune responses associated with the presence of nAbs, HAdV-based vectors may have limited appeal for use in clinical care (27). A growing alternative to circumvent this critical issue of pre-existing immunity is to substitute SAdVs for HAdVs (28). Although zoonotic, anthroponotic, and amphizoonosis events are possible (2, 29, 30), and have been documented between humans and non-human simian hosts (31 – 34), these appear to be uncommon. Additionally, it is reported that nAb prevalence against SAdVs is relatively low in human populations, when compared to HAdVs. For example, the seropositivity rates were 12.7% and 21% for SAdV-68 and SAdV-6, respectively (18, 26). The advantages of SAdVs and HAdVs as gene vectors include the capacity for large heterologous gene insertion, well-defined methods for preparation and purification, versatility for a wide host cell range (able to infect cells in mitotic and non-mitotic stages), and non-integration into the host genome. Adenovirus can recognize a variety of cell surface receptors, such as Coxsackie-adenovirus receptors (CARs) (35), CD46 (36), desmoglein-2 (DSG2) (37, 38), integrin (39), and so on. Both HAdVs and SAdVs have been genetically modified and used as delivery vehicles for gene therapy and vaccine applications, including Ebola and SARS-CoV-2, and also as oncolytic agents, e.g., for head and neck cancer and hepatocellular carcinoma (40 – 47).

In this report, a novel simian adenovirus vector was constructed and characterized in human cells to serve as a candidate gene therapy and vaccine vector, to bypass potential pre-existing immunity in humans. To accomplish this, a strategy of isolating novel SAdVs was undertaken: 115 samples of feces were collected at a zoological park; samples were screened for the presence of SAdVs using a generic primate adenovirus PCR assay; PCR-positive isolates were grown; and genomic DNA was isolated and characterized. From these, one isolate comprising an infectious adenovirus was isolated. The genome of this isolate GZ3-12 (GZ: Guangzhou; 3-12: sample number) was fully sequenced and annotated. Using the Gibson assembly method (48), a replication-competent infectious clone pBRSAdVGZ3-12 was firstly constructed and characterized. Subsequently, with this as a backbone, the E4orf6 gene was replaced with the HAdV-5 E4orf6 gene, then the E3 regions and E1B55K gene of this vector were deleted in order to construct a larger and presumably safer expression vector for ferrying exogenous genes. This recombinant vector includes and expresses enhanced Green Fluorescent Protein (eGFP) at high levels in A549, Caco-2, and Hela cells, for monitoring purposes. The availability of this novel replication-deficient SAdV vector provides an alternative and supports the further development of additional novel gene therapy and vaccine vectors.

## MATERIALS AND METHODS

### Cells, bacteria, viruses, plasmids, and enzymes

Macaque fecal samples were collected at a zoological park in Yunnan Province, China. Adenoviruses were isolated from these as described below. HEK 293 cells were purchased from the American Type Culture Collection (CBP60434). The cells were grown in Dulbecco’s modified Eagle’s medium (DMEM) plus 10% fetal calf serum (Gibco) at 37°C, in 5% CO_2_. *Escherichia coli* DH5α and *E. coli* BJ5183 competent cells were purchased from Stratagene (La Jolla CA, USA). Plasmid PBR322 and pEGFP C2 were purchased from TAKARA Co. (Dalian, China). Q5 DNA polymerase, T5 exonuclease, Phusion DNA polymerase, Taq DNA ligase, and restriction enzymes were from New England Biolabs (Beijing, China) and used according to the manufacturer’s instructions. Fluorescent quantitative PCR kits for HAdVs were purchased from Guangzhou Huayin Corp. (Guangzhou, China). The 5× isothermal reaction buffer used in the Gibson assembly method (48, 49) contained the following: 3 mL of 1 M Tris-HCl (pH 7.5), 150 µL of 2 M MgCl_2_, 600 µL of 10 mM dNTP, 300 µL of 1 M DTT, 1.5 g PEG-8000, and 300 mL of 100 mM NAD. Subsequent Gibson assembly master cocktails contained 40 µL 5× isothermal reaction buffer, 0.2 µL of 10 U µL^−1^ T5 exonuclease, 2.5 µL of 2 U µL^−1^ Phusion DNA polymerase, 20 µL of 40 U µL^−1^ Taq DNA ligase, and distilled water, to a final volume of 150 µL. All reagents were stored at −20°C.

### Adenoviral DNA extraction, PCR screening, and phylogenetic analysis

Approximately 200 mg of fecal sample was suspended with 1.6 mL absolute ethanol and centrifuged at 13,000 × *g* for 10 min at room temperature. The precipitate was washed two times with sterilized water to remove alcohol. About 300 µL of lysate buffer (4 M guanidine isothiocyanate and 1 mM EDTA) was added to the precipitate and incubated in a 56°C water bath for 10 min to lyse cells, and centrifuged at 14,000 × *g* for 10 min at room temperature. The upper solution was collected, and the viral nucleic acid was recovered with a PCR clean-up kit (AP-PCR-500, Axygen, USA), according to the manufacturer’s instructions.

PCR-based screening was performed using a primer pair targeting the highly conserved region of the hexon gene (Table S1) (50). PCR assays were performed using the TaKaRa Premix Taq Kit, using HAdV-3 strain GZ01 as the positive control (51). The PCR protocol consisted of a denaturation step at 94°C for 5 min, followed by 35 cycles of extension steps (94°C, 30 s; 55°C, 30 s; and 72°C, 20 s), and a final elongation step at 72°C for 10 min. PCR products were outsourced for DNA sequencing if the amplified band size was the same as that of the positive control.

### SAdV isolation, whole-genome sequencing, and annotation

About 200 mg of feces was washed with absolute ethanol and centrifuged at 13,000 × *g* for 10 min at room temperature for three times. Phosphate-buffered saline was added to the final precipitate, vortexed to resuspend, and then freeze/thawed using a −80°C freezer and a 37°C dry air incubator for three cycles (52). Samples were centrifuged at 12,000 × *g* for 10 min at room temperature, with the supernatants decanted and filtered through a 0.22-µm filter (Millipore). HEK293 cells, pre-grown on 60 mm plates, were inoculated with 500 µL of the filtrate and incubated for 2 h. Cells were then washed with fresh DMEM plus 2% fetal bovine serum, and antibiotics were added (100 U/mL of penicillin and 100 mg/mL of streptomycin) (53). When cytopathic effect (CPE) was present in more than 70% of cells, the infected cells and culture medium were subjected to freeze/thaw three times to release the virus. Viral genomic DNA was extracted and sent to a DNA sequencing core facility for Next-Generation whole-genome sequencing. Sequences were analyzed and compared to their corresponding reference genomes in GenBank and exons were annotated manually using SnapGene software (www.snapgene.com).

### Electron microscopy

HEK293 cells infected with SAdV GZ3-12 were harvested when CPE was observed in ≥90% of the cells. After centrifugation at 12,000 × *g* for 5 min, the supernatants were placed onto parafilm. A grid covered with carbon support Formvar film was floated on top of the virus suspension for 1 min, then it was put into sodium phosphotungstate (pH 7.0) and taken out to dry and exposed to UV irradiation for 20 min. Following this, the virus particles were observed under an electron microscope (FEI Tecnai-12, Hillsboro, OR, USA).

### Construction and verification of pBRSAdV GZ3-12 infectious clones

The Gibson assembly method provides for the easy and quick ligation of multiple DNA fragments *in vitro* through the overlapping regions of these fragments (49). We have successfully constructed an HAdV-14 infectious clone using this convenient and seamless splicing-based assembly method (54). Here, the complete genomic DNA of SAdV GZ3-12 was ligated with pBR322 to obtain an infectious clone using the Gibson assembly method, as shown in Fig. S1A. First, a novel adenovirus GZ3-12 was isolated from a fecal sample of a rhesus macaque as noted earlier; this was subsequently amplified in HEK293 cells. Viral genomic DNA was extracted from GZ3-12 using the modified extraction method described earlier by us (55). Separately, pBR322 was digested by *Eco*RI, with the linearized product serving as the PCR template. PCR amplification was performed with Q5 DNA polymerase (NEB) using a primer pair that contained 40 bp of the inverted terminal repeat (ITR) sequences of SAdV GZ3-12 and included *Swa*I site at both 5′ends (Table S1); *Swa*I is not found in the GZ3-12 genome. The resultant PCR product was digested with *Dpn*I enzyme to eliminate the plasmid template and purified subsequently. Then the SAdV GZ3-12 genomic DNA was added to this PCR product at a ratio of 4:1. Assembly master mixture was added for a total volume of 20 µL, and the entire reaction system was incubated at 50°C for 1 h. This resultant Gibson reaction product (final molecule shown in Fig. S1A) was transformed into *E. coli* DH5α competent cells and incubated on LB + ampicillin plates overnight. Two pairs of primers, which targeted the ligation sites of the two DNA fragments, were used in the PCR screening for positive clones using 2 µL of bacterium culture as PCR template. Briefly, primers SAdV-L-F and SAdV-L-R targeted the right end of pBR322 and the left terminal of inserted viral genome. Primers SAdV-R-F and SAdV-R-R targeted the left end of pBR322 and the right terminal of the viral genome. The PCR-positive plasmids were then extracted and their sizes were confirmed by gel electrophoresis. The positive plasmids were completely sequenced and then designated pBRSAdVGZ3-12.

### Construction of pSAdV-Ad5E4orf6, pSAdV-ΔE3-Ad5E4orf6-eGFP, pSAdV-ΔE3-eGFP, and pSAdV-ΔE1B55KΔE3-Ad5E4orf6-eGFP

To generate a plasmid containing both the SAdV genome and modified gene, a strategy was employed using the highly efficient homologous recombination system in *E. coli* BJ5183, as described earlier (56, 57). Two different prokaryotic host strains were used to produce and amplify recombinant adenoviruses plasmids. The first strain, BJ5183, is *rec*A proficient and supplies the machinery necessary to execute the recombination event between the shuttle vector and the pBRSAdVGZ3-12 vector. The second strain, *E. coli* DH5α, is used to amplify the recombined adenovirus plasmid. This strain is both endonuclease deficient (*endA1*) and recombination deficient (*recA*). The *endAl* mutation greatly improves the quality of plasmid miniprep DNA, and the *recA* mutation helps to insert stability (58, 59).

In order to improve the replication efficiency of the rescued viruses from the SAdV vector in human cells, HAdV-5 E4orf6 was added to the constructs, shown in Fig. S1C. First, HAdV-5 E4orf6 gene sequence was amplified by PCR (Fig. S1C, top right). In parallel, 2.5 kb of upstream and 900 bp of downstream sequences of the SAdV E4orf6 gene were also amplified. These three PCR segments (upstream, downstream, and HAdV-5 E4orf6) were inserted into a pUC19 vector to construct the pUC-Ad5E4orf6 shuttle plasmid, using a ClonExpress MultiS One Step Cloning Kit (Vazyme, Nanjing, China), according to the manufacturer’s instructions. As shown in the top left of Fig. S1C, pSAdVGZ3-12 was then digested with *Spe*I (releasing the viral genomic DNA), and pUC-Ad5E4orf6 shuttle plasmid was digested with *Bam*HI and *Rsr*II. About 200 ng each of the two nucleic acid fragments was simultaneously added to the BJ5183 competent bacteria for homologous recombination in order to replace SAdV E4orf6 with HAdV-5 E4orf6. The resultant vector was designated pSAdV-Ad5E4orf6 (Fig. S1C, middle).

In order to increase the capacity of the vector for large heterologous inserts, the E3 regions of both pBRSAdVGZ3-12 and pSAdV-Ad5E4orf6 were deleted by digestion with *Pac*I (Fig. S1B and C, respectively) and an eGFP gene which is driven by CMV promotor was inserted (Fig. S1B and C). PCR was performed to amplify the upstream and downstream sequences of the E3 region from SAdV GZ3-12. In addition, a CMV-eGFP-SV40 expression cassette was amplified by PCR using pEGFP-C2 plasmid as template (56). About 15–20 bp of overlapping sequences was designed at each end of the three fragments to serve as targets for recognition and ligation. The three fragments were then assembled into a new shuttle plasmid named pUC-ΔE3eGFP, according to the manufacturer’s instructions for the ClonExpress MultiS One Step Cloning Kit (Vazyme). Homologous recombination resulted in the replacement of the E3 region of pBRSAdVGZ3-12 and pSAdV-Ad5E4orf6 by eGFP. This constructed vector was named pSAdV-ΔE3-eGFP and pSAdV-ΔE3-Ad5E4orf6-eGFP.

In order to further increase the capacity of the vector for large heterologous inserts, the SAdV E1B55K gene of pSAdV-ΔE3-Ad5E4orf6-eGFP was deleted by digestion with *Pme*I, as shown in Fig. S1C, middle lower portion. The PCR amplified 737 bp of upstream sequences and 1,314 bp of downstream sequence of SAdV E1B19K gene. Following this, the two PCR fragments with the linearized pUC19 vector were ligated according to the ClonExpress MultiS One Step Cloning Kit. The shuttle vector and pSAdV-ΔE3-E4orf6-eGFP were homologous-recombined in BJ5183 competent cells, resulting in the vector lacking E1B55K, named pSAdV-ΔE1B55KΔE3-Ad5E4orf6-eGFP.

### Construction of E3 complementing HEK293-E3 cells

In order to construct HEK293 cells that express the E3 region of GZ3-12, the SAdV E3 region was amplified using plenti-E3F/R primers, and cloned into pLenti vector, named pLenti-E3. Then, HEK293 cells were cultured in six-well plates until they reached 80% confluence. The pLenti-E3, pMD2.G, and pSPAX2 were co-transfected into HEK293 cells at a ratio of 1:4:3 according to the instructions of Lipofectamine3000 Transfection Kit (60). At the same time, the pLenti-Empty, pMD2.G, and pSPAX2 were co-transfected into HEK293 cells at the same ratio and served as negative control. The medium was changed 12 h after transfection. Thirty-six hours and 60 h post-transfection, cell supernatants containing lentivirus were collected and filtrated with 0.45 µm filter. The harvested virus culture was used to infect HEK293 again. At 48 h post-infection (hpi), the cells were selected with puromycin (Sigma, P8833) for 4–7 days. The expression of E3 region genes was determined by reverse transcription PCR. The constructed cell lines were named HEK293-E3 and HEK293-Empty, respectively.

### Rescue of SAdV GZ3-12, SAdV-Ad5E4orf6, SAdV-ΔE3-Ad5E4orf6-eGFP, and SAdV-ΔE1B55KΔE3-Ad5E4orf6-eGFP infectious viral particles

The constructed plasmid vectors were digested by *Swa*I restriction endonuclease to release the SAdV genomes and subsequently transfected into HEK293 cells using Lipofectamine 3000 (Invitrogen, Carlsbad, CA, USA) according to the manufacturer’s instructions. When CPE was observed in at least half of the cells, the cultures were subjected to three rounds of freeze/thaw. The harvested supernatants were then used to infect fresh HEK293 cells. When CPE was observed in half of these cells, the culture was harvested again and stored at −80°C. Rescued viruses were named SAdV GZ3-12, SAdV-Ad5E4orf6, SAdV-ΔE3-Ad5E4orf6-eGFP, and SAdV-ΔE1B55KΔE3-Ad5E4orf6-eGFP.

### Restriction enzyme analysis and DNA sequencing analysis of plasmid vectors pBRSAdVGZ3-12, pSAdV-Ad5E4orf6, pSAdV-ΔE3-Ad5E4orf6-eGFP, and pSAdV-ΔE3ΔE1B55K-Ad5E4orf6-eGFP and the rescued viruses

To determine if any deletion/insertion (indels) mutations occurred during the plasmid vector construction and viral rescue procedures, restriction enzyme analysis of vectors pBRSAdVGZ3-12, pSAdV-Ad5E4orf6, pSAdV-ΔE3-Ad5E4orf6-eGFP, and SAdV-ΔE1B55KΔE3-Ad5E4orf6-eGFP, as well as the rescued viral genomic DNA, was performed. The *in silico* digestion maps were produced by SnapGene software (www.snapgene.com). The plasmids were digested with five restriction endonucleases, *Eco*RI, *Kpn*I, *Eco*RV, *Not*I, and *Bam*HI. The products were analyzed by agarose gel electrophoresis. Rescued viral genomic DNA was also digested with the same restriction endonucleases. The complete nucleotide sequences of the plasmids were determined by the Sanger chemistry primer-walking method, with an average of three- to fivefold coverage. The assembled sequences were aligned with either the SAdV GZ3-12 genomic sequence or the pBR322 sequence, to assess if mutations were generated inadvertently during the construction.

### Microneutralization assays

CPE-based microneutralization (NT) assays were performed using HEK293 cells on 96-well microtiter plates as previously described (46, 61). Briefly, the cells were seeded 1 day before infection. Before assay, the human sera were heat-inactivated at 56°C for 30 min. Twofold serial dilutions ranging from 1:4 to 1:512 of the serum samples were incubated with 200 TCID_50_ of GZ3-12, and placed in the incubator at 37°C for 1 h. Afterward, 100 µL of the incubated serum and virus mixture was added to each well, and the plates were placed back into the incubator for 2 h. In the final step, the mix of sera and viruses was removed and replaced with DMEM containing 2% fetal bovine serum. HAdV-5 was included as a positive control. On the fifth day after infection, the CPE in cells was examined and photographed, and the antibody titer was calculated using Reed-Muench method (62). All of the experimental protocols for human sera in this study were approved by the Medical Ethics Board of Southern Medical University, and carried out in accordance with the Declaration of Helsinki, as revised in 2013. Data records of the samples and sample collection are de-identified and completely anonymous.

### One-step growth curves of SAdV GZ3-12, SAdV-Ad5E4orf6, SAdV-ΔE3-Ad5E4orf6-eGFP, and SAdV-ΔE1B55KΔE3-Ad5E4orf6-eGFP

To assess the viral replication efficiency of SAdV-Ad5E4orf6, SAdV-ΔE3-Ad5E4orf6-eGFP, and SAdV-ΔE1B55KΔE3-Ad5E4orf6-eGFP, quantitative PCR (qPCR) was performed using SYBGreen real-time PCR with hexon-based plasmids as standards, as previously described (48). HEK293 cells were seeded onto 12-well plates and inoculated with SAdV at a multiplicity of infection (MOI) of 0.5. Aliquots were harvested every 12 h post-infection, and the viral genomic DNA copy numbers were quantified via qPCR. Data analysis was performed with the QuantStudio Real-Time PCR Software (Thermo Fisher Scientific, USA). One-step growth curves were drawn using GraphPad Prism V6.0 (GraphPad Software Inc., San Diego, CA, USA).

### siRNA assay

Three pairs of siRNAs targeting hCD46, hCAR, and hDSG2 receptors were synthesized by Sangon Biotech (Shanghai, China). These three pairs of siRNAs have been demonstrated efficiently in previous studies to knock down the expression of the three receptors in A549 cells (37). The sequences of siRNA are shown in Table 1. siRNA transfection was performed using Lipofectamine 3000 Transfection Reagent (Thermo Fisher, Shanghai, China). A total of 1 × 10^4^ A549 cells were transfected with 1 µg of siRNA for hDSG2, hCAR, hCD46, or scarmble siRNA, respectively. Forty-eight hours after siRNA transfection, the cells were infected with 1 MOI of SAdV GZ3-12. HAdV-3, HAdV-5, and HAdV-55 were used as positive control. At 48 hpi, the viral genomic DNA copy numbers in the cells were quantified via qPCR.

**TABLE 1:** Percent identities of the nucleotide sequences of selected coding regions from SAdV GZ3-12 and three phylogenetically related SAdV genomes^*a*^

Region	Genome	L3	L2	L5	E1A	E1B	IX	L1	L3	E4			
Position	1–34,094	17,776–20,529	29,497–31,098	29,497–31,099	491–1,354	1,516–2,073	3,432–3,842	10,405–11,505	16,918–17,688	33,355–33,741	31,414–32,268	32,198–32,563	32,582–32,929
CDS	**/**	Hexon	Penton base	Fiber	E1A	E1B 19K	Protein IX	52K	pVI	E4orf1	E4orf6	E4orf4	E4orf3
SAdV-3	90.5%	82.61%	93.40%	75.26%	97.46%	97.13%	**98.78%**	**96.39%**	**97.15%**	**93.02%**	**95.20%**	**97.54%**	**92.82%**
SAdV-6	**95.5%**	**96.84%**	**96.30%**	**85.46%**	96.08%	97.13%	98.05%	96.22%	96.76%	89.66%	91.70%	90.16%	89.66%
SAdV-48	88.1%	79.65%	91.22%	72.22%	**97.92%**	**97.85%**	98.05%	95.30%	95.72%	89.66%	92.40%	96.72%	90.23%

^*a*^ The numbers in bold are the highest percent identities. CDS positions are relative to SAdV GZ3-12.

### Sequence data and statistical analysis

Genome sequence data for the following viruses were accessed from GenBank.

HAdV-4, AB330085.1; HAdV-40, AB330121.1; HAdV-41, AB330122.1; HAdV-52, DQ923122.2; HAdV-3, AB330084.1; HAdV-18, AB330099.1; HAdV-11, AB330092.1; HAdV-12, AB330093.1; HAdV-7, AB330088.1; HAdV-1, AB330082.1; HAdV-2, AB330083.1; HAdV-50, AB330131.1; HAdV-44, AB330125.1; HAdV-43, AB330124.1; HAdV-8, AB330089.1; HAdV-49, AB330130.1; HAdV-9, AB330090.1; HAdV-51, AB330132.1; HAdV-31, AB330112.1; HAdV-61, KX868299.1; HAdV-52, DQ923122.2; HAdV-56, LC215445.1; HAdV-57, KF835458.1; A1285, JN880452.1; A1258, JN880451.1; A1173, JN880450.1; SAdV-7, DQ792570.1; SAdV-45, FJ025901.1; SAdV-43, FJ025900.1; SAdV-13, KT984509.1; SAdV-50, HQ241820.1; SAdV-DM-2014, KM190146.1; SAdV-25, AC_000011.1; SAdV-3, NC_006144.1; SAdV-24, AY530878.1; SAdV-16, NC_028105.1; SAdV-18, NC_022266.1; SAdV-20, NC_020485.1; SAdV-49, NC_015225.1; SAdV-60, MF198454.1; SAdV-6, JQ776547.1; SAdV-48, HQ241818.1; SAdV-40.2, FJ025926.1; BaAdV-2, NC_021168.1; BaAdV-4, KC693024.1; BaAdV-3, KC693023.1; SAdV-26, FJ025923.1; SAdV-37.1, FJ025921.1; SAdV-29, FJ025916.1; and SAdV-33, FJ025908.1.

Single sequence alignments were performed using the NCBI nucleotide blast module. Multiple sequence alignments were generated using tools in the software Multiple Alignment using Fast Fourier Transform package (MAFFT version 7), with default parameters (https://mafft.cbrc.jp/alignment/server/). MAFFT was selected for all alignments, including gene sequences, due to its speed and its capacity to handle a large amount of data. And phylogenetic trees were constructed using MEGA 11 software (63) using the maximum-likelihood method with default parameters and 1,000 replications. Bootstrap values were noted on the branches and at nodes.

The package of SPSS Statistics 20.0 was used for analysis of the experimental data, and GraphPad 8.0 was used to record the results. Two or more sample rates are tested using chi-square tests, and multiple samples were compared using one-way ANOVA. Data from three independent experiments were obtained, with mean ± SD noted, and *, *P*＜0.05, **, *P*＜0.01, ***, *P*＜0.001, and ****, *P*＜0.0001.

## RESULTS

### Identification of SAdV isolates by PCR, genome sequencing, and phylogenetic analysis

**Fig 1:**
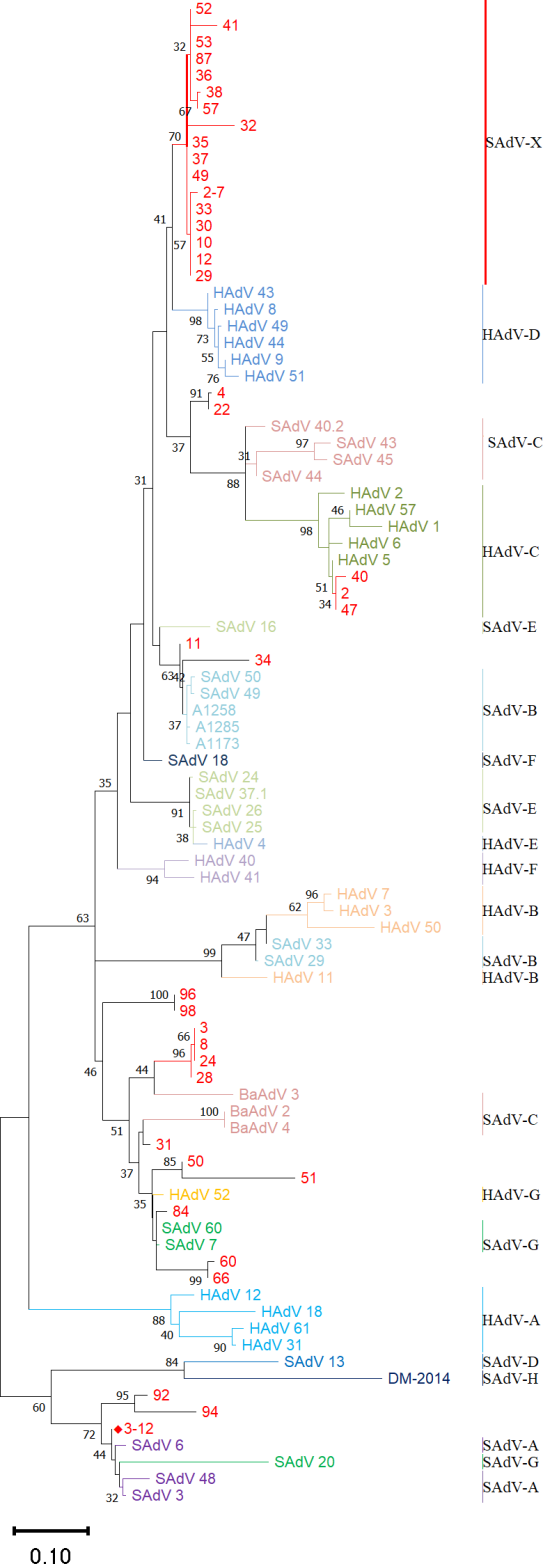
Phylogenetic distribution of SAdVs obtained from simian fecal samples. Phylogenetic trees based on partial hexon gene were generated using the maximum-likelihood method and applying default parameters with 1,000 replications. Bootstrap values are noted on the branches, with 80 considered robust. Numbers on the leaves represent the sample number from which the sequence was obtained. Select HAdVs and SAdVs are included for reference. The sequences noted in red correspond to samples obtained in this study. GZ3-12 is noted by a red, solid diamond shape, and is the virus that was isolated, characterized, and served as the basis for subsequent vector development.

To isolate and identify SAdVs from macaques, 115 fecal samples were screened, with 46 samples testing positive by PCR using primers targeting the highly conserved region of the hexon gene (Table S1). Among these, 33.9% (39/115) sequences were recovered from these PCR^+^ samples, while 7 of the 46 samples did not generate sequences. Using BLAST, 48.7% (19/39) of these sequences had high percent identities with SAdV sequences archived in GenBank. Additionally, 51.3% of the sequences showed sequence similarities to HAdVs. To explore the phylogenetic relationship of these sequences, multiple sequence alignments were performed, followed by phylogenetic analysis using the maximum-likelihood algorithm. Using reference sequences from HAdVs and SAdVs (HAdV species A–G and SAdV species A–G representatives), a phylogenetic tree was constructed by MEGA 11 (Fig. 1). The 39 sequences were parsed into six species (HAdV species B, C, D and SAdV species A, B, G). The largest number of these isolated AdVs were classified as similar to HAdV species D genotypes (43.6%; 17/39). This result indicates that there may either be some SAdVs (SAdV-X) that are closely related to HAdVs in wild macaque populations, or zoonosis and anthroponosis had occurred among the two AdV groups in the wild as well as in captive animal settings such as zoological parks and primate research centers (29, 30, 32).

### Virus isolation, whole-genome sequencing, and phylogenetic analysis

**Fig 2:**
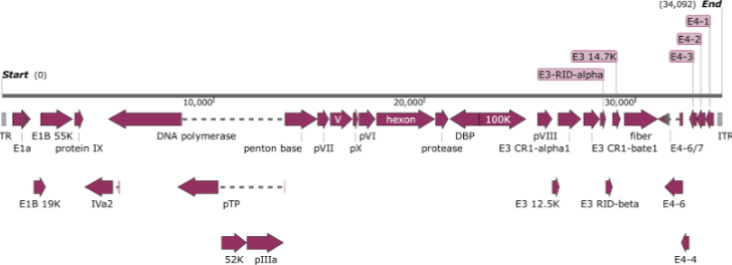
Annotation of SAdV GZ3-12 genome. Protein names are noted, as homologs of SAdV-6 counterparts. Transcriptional direction is noted by arrows.

SAdV GZ3-12 was selected, cultured, and isolated because it appeared preliminarily to be a SAdV species A member (Fig. 1), and unlikely to have previously infected a substantial proportion of the human population. Based on phylogenetic data, it is most distant from the HAdVs. Genomic DNA from SAdV GZ3-12 was extracted and sequenced. This genome was annotated using SAdV-6 as a reference (GenBank accession number JQ776547). The genome comprises 34,094 bp with a GC content of 55.6%, and ITR sequences noted of 150 bp each. There are 30 putative coding regions (Fig. 2), which is characteristic for mastadenoviruses.

**Fig 3:**
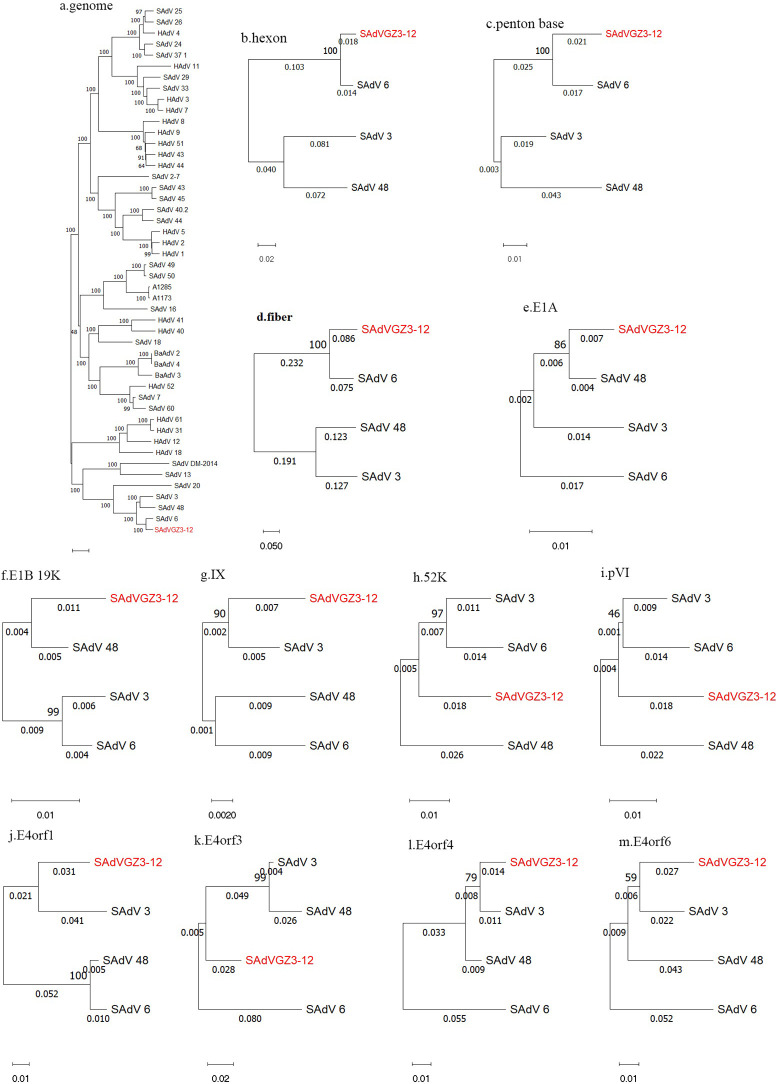
Phylogenetic analysis of whole-genome sequences and select genes of SAdV GZ3-12. The DNA sequences of the whole genome and 10 genes spanning the genome are presented. From the whole-genome analysis, SAdV GZ3-12 is determined to be a novel member of SAdV species A, forming a subclade with SAdV-3, -6, and -48. Closer inspections of the 10 genes from members of this subclade are presented. Phylogenetic trees were generated using the maximum-likelihood method and by applying default parameters with 1,000 replications. The sequences include the following: whole genome, hexon, penton base, fiber, E1A, 19 kDa protein, pIX, 52 kDa protein, pVI, E4orf1, E4orf3, E4orf4, and E4orf6.

As noted in Fig. 3a, SAdV GZ3-12 forms a subclade with members of the SAdV species A. Whole-genome comparisons of SAdV GZ3-12 show it is most similar to the genome of SAdV-6 (95.5%), forming a subclade. However, sequence percent identity analysis of coding regions reveals that 9 coding regions spanning the genome in SAdV GZ3-12, out of 12 examined, are more similar to counterparts from other members of SAdV species A (Tables 1 and 2). Of these SAdVs, the penton base and hexon genes have the highest sequence similarities with their SAdV-6 counterparts. This is also observed for the other genes examined. However, the fiber gene and protein of SAdV GZ3-12 have relatively low sequence similarities with their SAdV-six counterparts (85.46% and 88.18%, respectively). The phylogenetic analyses of select 12 genes of SAdV GZ3-12 (Fig. 3) show matching sequence similarities with SAdV-3, -6, and -48 (Tables 1 and 2). Therefore, these analyses indicate that SAdV GZ3-12 is a novel SAdV isolate that is distinct from SAdV-6 and other SAdV species A members.

**TABLE 2:** Percent identities of selected amino acid sequences from SAdV GZ3-12 and three phylogenetically related SAdV genomes^*a*^

Region	L3	L2	L5	E1A	E1B	IX	L1	L3	E4			
Position	17,776–20,529	29,497–31,098	29,497–31,099	491–1,354	1,516–2,073	3,432–3,842	10,405–11,505	16,918–17,688	33,355–33,741	31,414–32,268	32,198–32,563	32,582–32,929
CDS	Hexon	Penton base	Fiber	E1A	E1B 19K	Protein IX	52K	pVI	E4orf1	E4orf6	E4orf4	E4orf3
SAdV-3	84.22%	96.03%	50.62%	**98.08%**	**98.78%**	**99.26%**	98.67%	98.05%	**98.33%**	**96.83%**	98.20%	90.43%
SAdV-6	**99.78%**	**99.01%**	**88.18%**	96.17%	98.17%	**99.26%**	99.20%	97.66%	96.88%	93.96%	91.81%	**95.65%**
SAdV-48	86.25%	96.03%	50.44%	94.78%	93.90%	**99.26%**	**99.73%**	**98.83%**	95.31%	96.13%	**100%**	90.5%

^*a*^ The numbers in bold are the highest percent identities. CDS positions are relative to SAdV GZ3-12.

### Electron microscopy

**Fig 4:**
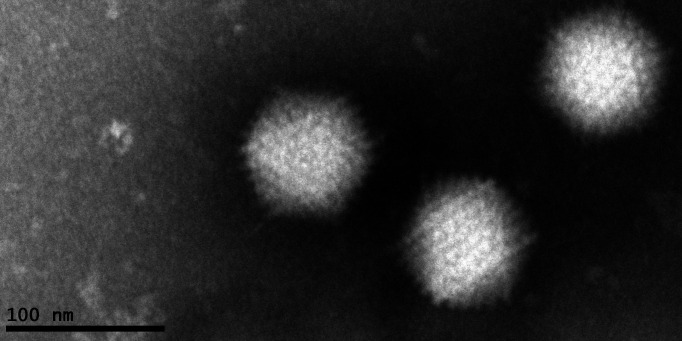
Electron micrograph visualization of SAdV GZ3-12. Its diameter is approximately 70–90 nm, consistent with human adenoviruses. Magnification 43,000×. Scale bar: 100 nm.

At magnification 98,000× under the electron microscope (FEI, Tecnai G^2^ 20 TWIN, 200 kV), typical adenovirus particles of SAdV GZ3-12 were observed with a diameter of about 70–90 nm (Fig. 4).

### Seroprevalence of neutralizing antibodies against SAdV GZ3-12

To survey the seroprevalence of neutralizing antibodies against GZ3-12 in the human population, 300 human serum samples were collected from several geographical locations, including six provinces in China. Healthy donors and their sera were randomly chosen for inclusion in the study. In this study, 53.3% were from males and 46.7% were from females. Microneutralization tests were performed on these serum samples. Thirty out of 300 (10.0%) were assessed as positive for GZ3-12 neutralizing antibodies (titer > 18). No serum had a neutralization titer higher than 64. It should be noted that no serum showed strong seropositivity against GZ3-12 as judged by serum neutralizing antibody titers: negative (<1:18), low (1:18 to 1:200), moderate (1:200 to 1:1,000), and high (>1:1,000) (61, 64). There were no significant differences in the SAdV-seropositive rates by gender (*χ* ^2^-test, *P* value = 0.885): 9.4% for males and 10.7% for females. At the same time, the seroprevalence was also not statistically different among provinces (*χ* ^2^-test, *P* value = 0.822): 10.0% for Hebei, Hunan, and Jiangsu provinces, 15.0% for Shannxi and Shandong provinces, and 9.0% for Guangdong Province. This result indicates that the serum neutralizing antibody level against SAdV GZ3-12 in healthy people is very low. In contrast, the neutralizing antibody titers against HAdV-C5 detected simultaneously were all positive and greater than 1:256 in randomly selected 30 serum samples. Additionally, in our earlier investigation of HAdV-5 seroprevalence in Guangzhou, China, we found that the seroprevalence was 77.34% in the general healthy population (65). The proportion of neutralizing antibody titers is 22.66%, 13.31%, 18.35%, and 45.68% for titers <1:18, 1:18 to 1:200, 1:200 to 1:1,000, and higher than 1:1,000, respectively (65).

### Identification of a pBRSAdVGZ3-12 infectious clone and rescue of viral particles

Infectious clones of plasmid pBRSAdVGZ3-12 were generated by the ligation of SAdV GZ3-12 genomic DNA with the PCR amplicon of linearized pBR322 (Fig. S1A). Fifteen colonies were selected and assayed by PCR, of which 2 were identified as PCR-positive. These two constructs were further characterized to identify any unintentional nt insertions/deletions (indels) or mutations by sequencing the whole plasmids. Sequencing did not reveal any indels or mutations in the constructs.

**Fig 5:**
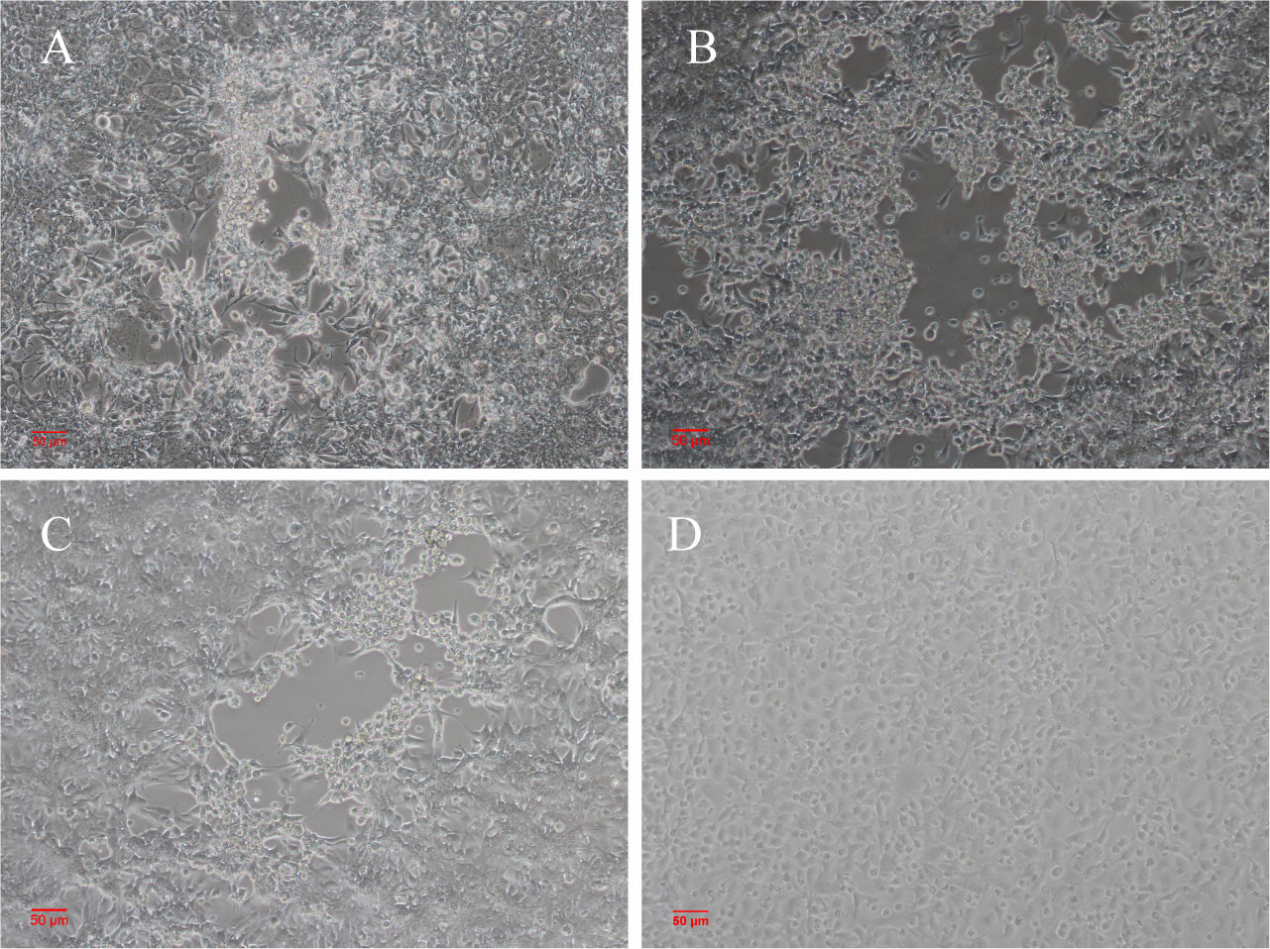
CPE observed in HEK293 cells at day 5 post-transfection or infection. (**A**) HEK293 cells transfected with pBRSAdVGZ3-12-derived adenovirus genomic DNA. (**B**) HEK293 cells infected with one of the two pBRSAdVGZ3-12-derived adenvoiruses. (**C**) HEK293 cells infected with SAdV GZ3-12. (**D**) Normal HEK293 cells. There was no obvious difference in CPE.

The linearized SAdV GZ3-12 genome was released by S*wa*I digestion of the pBRSAdVGZ3-12 infectious clone, and transfected onto HEK293 cells. After 96 h of transfection, the culture was frozen and thawed three times, and the supernatant was recovered and used to re-infect HEK293 cells. At day 5 post-infection, CPE was observed for both infection by control SAdV GZ3-12 and the transfection of the experimental pBRSAdVGZ3-12 constructs, as shown in Fig. 5. This result indicates that the constructed SAdV infectious clone can rescue infectious virus particles and can be used for further virus modification.

### Vector identification and viral rescue of pSAdV-Ad5E4orf6, pSAdV-ΔE3-Ad5E4orf6-eGFP, and SAdV-ΔE1B55KΔE3-Ad5E4orf6-eGFP

**Fig 6:**
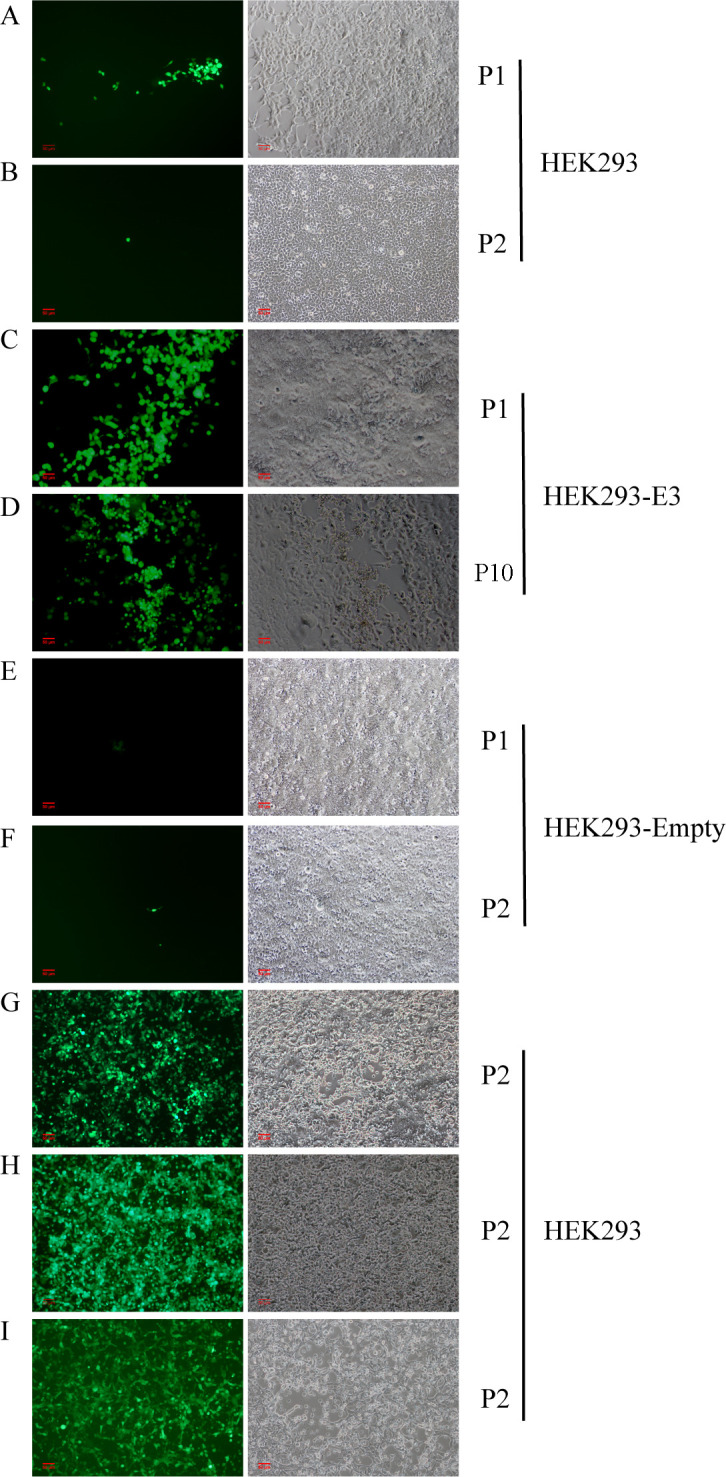
HEK293 and HEK293-E3 cells infected by E1/E3-deficient SAdVs. (**A and B**) The first generation (P1) of SAdV-ΔE3-eGFP infected HEK239 cells could express a small amount of GFP for only 5 days, then the GFP disappeared. When the culture from P1 was used to infect HEK293 cells, no GFP was observed (P2). (**C and D**) SAdV-ΔE3-eGFP could be rescued in HEK293-E3 cells and cause CPE (P1), and a large amount of GFP could still be observed even after 10 generations (P10) of virus culture. (**E and F**) The first generation (P1) of SAdV-ΔE3-eGFP-infected HEK239-Empty cells could express a small amount of GFP for only 5 days, then the GFP disappeared. When the culture from P1 infected HEK293-Empty cells, no GFP was observed (P2). (**G**) HEK293 cells infected by SAdV-ΔE3-Ad5E4orf6-eGFP. Expression of GFP was observed under the inverted fluorescence microscope at day 6 post-infection. (**H**) HEK293 cells infected by SAdV-ΔE3ΔE1B55K-Ad5E4orf6-eGFP. Expression of GFP was observed under the inverted fluorescence microscope at day 6 post-infection. (**I**) HEK293 cells infected by HAdV-5-GFPΔE1ΔE3, HAdV-5-GFP was used as a positive control in this study. The magnification of all images is 100×.

In order to verify whether the virus could produce progeny viruses in the absence of the E3 region, we deleted the E3 region of the adenovirus vector and inserted an eGFP gene to serve as an expression reporter (Fig. S1B). The genome was released from the constructed clone by *Swa*I digestion, and then transfected into HEK293 cells. On the sixth day post-transfection, a cluster of cells expressing GFP was observed. Then the culture was harvested, and used to infect HEK293 cells. Green fluorescence was also observed (Fig. 6A). It was noted that the fluorescence was observable for only 5 days, then it disappeared. The culture from P1 was harvested and infected HEK293 again. Interestingly, no green fluorescence was observed (Fig. 6B). Furthermore, nucleic acid of the adenovirus was undetectable in the P2 culture. This suggested that the adenovirus with E3-deletion does not produce infectious progeny in HEK293 cells. However, when the SAdV genomic DNA released from pSAdV-ΔE3-eGFP was transfected and cultured in HEK293-E3 cells stably expressing the SAdV E3 proteins, green fluorescence could be observed (P1 viruses) (Fig. 6C). Even after passaging in HEK293-E3 cells for 10 generations, green fluorescence and CPE could still be observed in the infected cells (Fig. 6D). Additionally, the SAdV nucleic acid in the culture can also be detected by PCR from generations 1 to 10. However, the replication of the virus in HEK-Empty cells is consistent with that in HEK293 cells (Fig. 6E and F). This supports the hypothesis that the E3 region is necessary for SADV GZ3-12 replication in HEK293 cells.

Studies have shown that E4orf6 of HAdV-5 can improve the replication efficiency of adenoviruses in HEK293 cells (66). With reference to this, we replaced the SAdV GZ3-12 E4orf6 with Ad5-E4orf6, and rescued the resultant SAdV-Ad5E4orf6 construct. This construct produced CPE in HEK293 cells similar to the wild-type virus. Then, the E3 region of pSAdV-Ad5E4orf6 was deleted and replaced by eGFP, and the construct SAdV-ΔE3-Ad5E4orf6-eGFP was rescued in HEK293 cells, which could continuously express green fluorescence and cause CPE (Fig. 6G), suggesting that SAdV-ΔE3-Ad5E4orf6-eGFP could replicate well in HEK293 cells and Ad5E4orf6 could complement E3 region function.

In addition, SAdV E1B55K was deleted and the resultant construct named SAdV-ΔE3ΔE1B55K-Ad5E4orf6-eGFP was successfully rescued. Both viral DNA (data not shown) and green fluorescence were detected in subsequent cultures (Fig. 6H). HAdV-5-GFPΔE1ΔE3 was also constructed by AdEasy Adenoviral Vector System and used as a positive control in this study (Fig. 6I). The rescued adenoviruses were passaged in HEK293 cells, which express HAdV-5 E1A and E1B, for 20 generations. No reverse mutations were observed in the E1, E3, and E4 regions, indicating stable adenoviral genomes. Infection of A549 cells which do not express HAdV-5 E1 genes with the 21st generation of virus cultures showed no infectious progeny viruses were produced. Even if the virus culture harvested from A549 cells was used to infect A549 cells and cultured for more than 5 days, the adenoviral DNA could not be detected, which indicated that SAdV-ΔE3-Ad5E4orf6-eGFP and SAdV-ΔE3ΔE1B55K-Ad5E4orf6-eGFP were stable during passages in HEK 293 cells (expressing HAdV-5 E1B55K), but no replication-competent adenovirus capable of replicating in noncomplementing cells was produced. HAdV-5 E4orf6 can complement E3 region function of SAdV and improve the replication efficiency of SAdV-ΔE3-Ad5E4orf6-eGFP.

### Sequence validation of constructed vectors and rescued viruses by restriction endonuclease analysis

**Fig 7:**
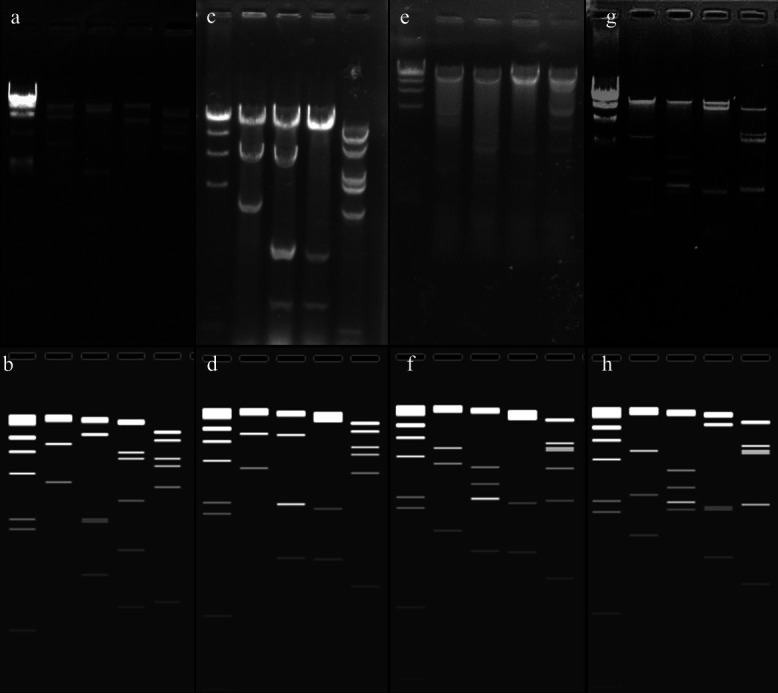
Restriction enzyme digest characterization of the constructs. Genomes of SAdV GZ3-12 (panels (A) and (B)), SAdV-Ad5E4orf6 (panels (C) and (D)), SAdV-ΔE3-Ad5E4orf6-eGFP (panels (E) and (F)), and SAdV-ΔE3ΔE1B55K-Ad5E4orf6-eGFP (panels (G) and (H)) were digested with restriction enzymes and electrophoresed. Lane 1: lambda DNA/H*ind*III Marker; lane 2: DNA/*Eco*RI; lane 3: DNA/*Kpn*I; lane 4: DNA/*Not*I (A–F) or *Eco*RV (G–H); and lane 5: DNA/*Bam*HI. The experimental RE digests are shown in the top panels (A, C, E, G) and the computed *in silico* patterns are displayed in the bottom panels (B, D, F, H) (www.snapgene.com). No indels occurred during homologous recombination.

In order to verify that there were no insertions or deletions (indels) in the constructs, seven pairs of primers were designed to amplify across the genome. We identified no indels in the amplified regions by this PCR analysis (data not shown). As an additional assay, genomic DNA of the rescued viruses from pSAdV-Ad5E4orf6, pSAdV-ΔE3-Ad5E4orf6-eGFP, and SAdV-ΔE1B55KΔE3-Ad5E4orf6-eGFP were digested by restriction enzymes *in vitro* and *in silico*. The *in vitro* restriction enzyme digest profiles of the constructed plasmids and the genome of rescued viruses (Fig. 7) were consistent with the *in silico* digestion maps produced by SnapGene software (www.snapgene.com). These results indicate that no indels occurred during the homologous recombination process.

### The infectivity of SAdV GZ3-12 in four human cell lines

**Fig 8:**
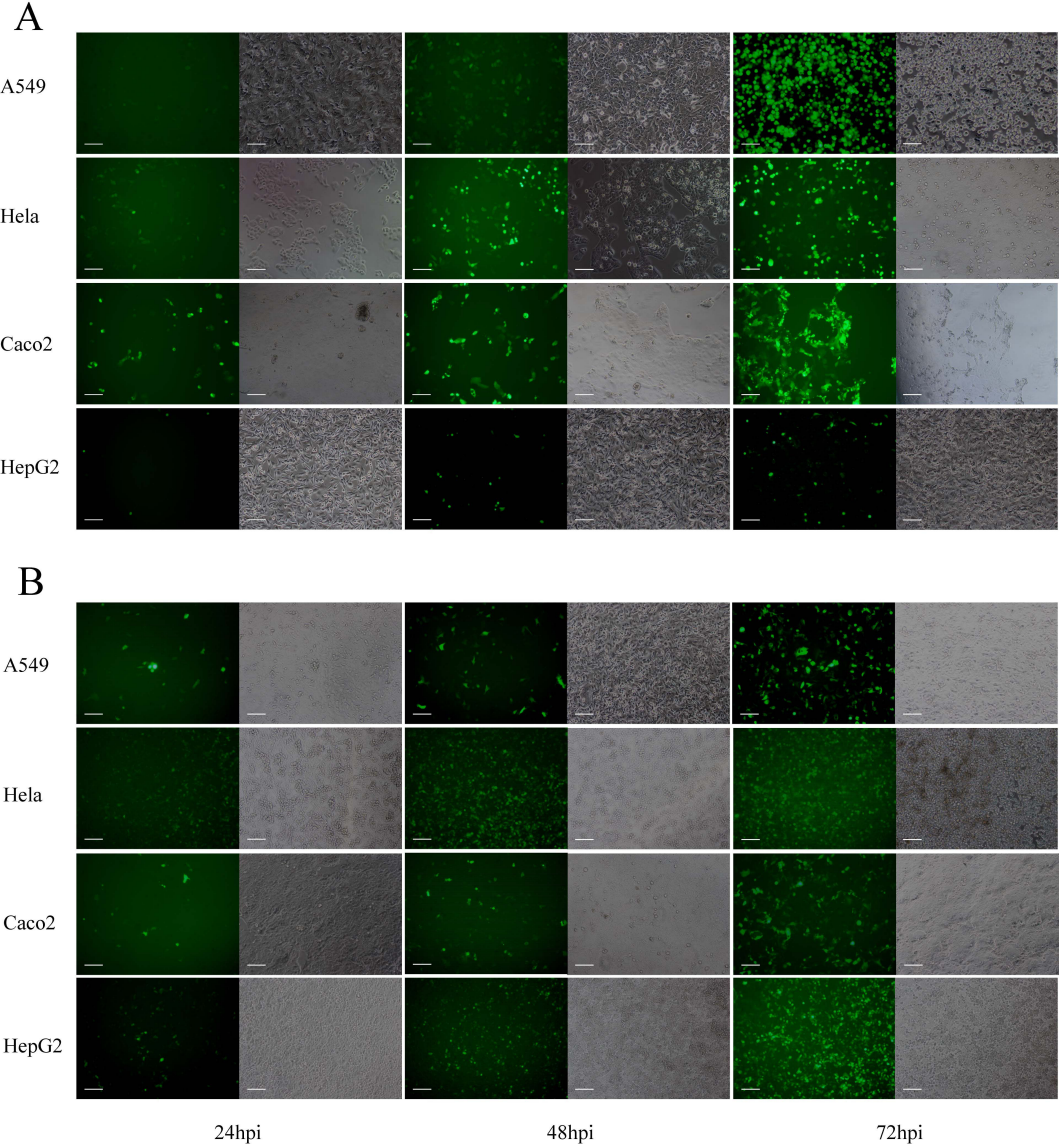
SAdV GZ3-12 and HAdV-5-GFP infections in four human cancer cell lines. A549, HeLa, Caco2, and HepG2 cells were infected with SAdV-ΔE3ΔE1B55K-Ad5E4orf6-eGFP (**A**) or HAdV-5-GFP (**B**) at a multiplicity of infection (MOI) of 1. Infectivity was assessed at 24, 48, and 72 h post-infection (hpi) by the observation of GFP expression under a fluorescence microscope at 100× magnification. Scale bar: 100 µm.

The ability of SAdV GZ3-12 to infect four human cell lines was assessed. These included A549, HeLa, Caco2, and HepG2 cells. Cells were aliquoted into 12-well plates 1 day before infection and placed in an incubator. They were then infected with SAdV-ΔE3ΔE1B55K-Ad5E4orf6-eGFP at an MOI of 0.5. The infections were followed and documented at 24, 48, and 72 hpi (Fig. 8). As noted, SAdV-ΔE3ΔE1B55K-Ad5E4orf6-eGFP infected the four cell lines, as eGFP expression was observed within 24 h in all lines. However, there were differences in the eGFP expression levels amongst the four cell lines, with higher expression in A549, Caco2, and HeLa cells, with relatively less expression in HepG2 cells (Fig. 8A). Using HAdV-5 at the same MOI to infect cells, it was found that HAdV-5 had a higher infection efficiency on Hela and HepG2 cells, but a lower infection efficiency on A549 and Caco2 cells (Fig. 8B). These results show that GZ3-12 can infect a variety of human cancer cell lines, but the infection efficiency and heterologous gene expression levels differed. The supernatants from these viral cultures were harvested and used to infect the same cell lines, and no eGFP expression was observed at 24, 48, and 72 h (data not shown). SAdV genome was also undetectable by qPCR using universal primers hexon F/R after these reinfections (data not shown). This indicates that the rescued simian adenoviruses with the deletion of E1B55K, E3 region, and E4orf6 genes can infect various cancer cells and express foreign genes, but cannot produce infectious virions.

### One-step growth curve comparisons between SAdV GZ3-12 and the constructs

**Fig 9:**
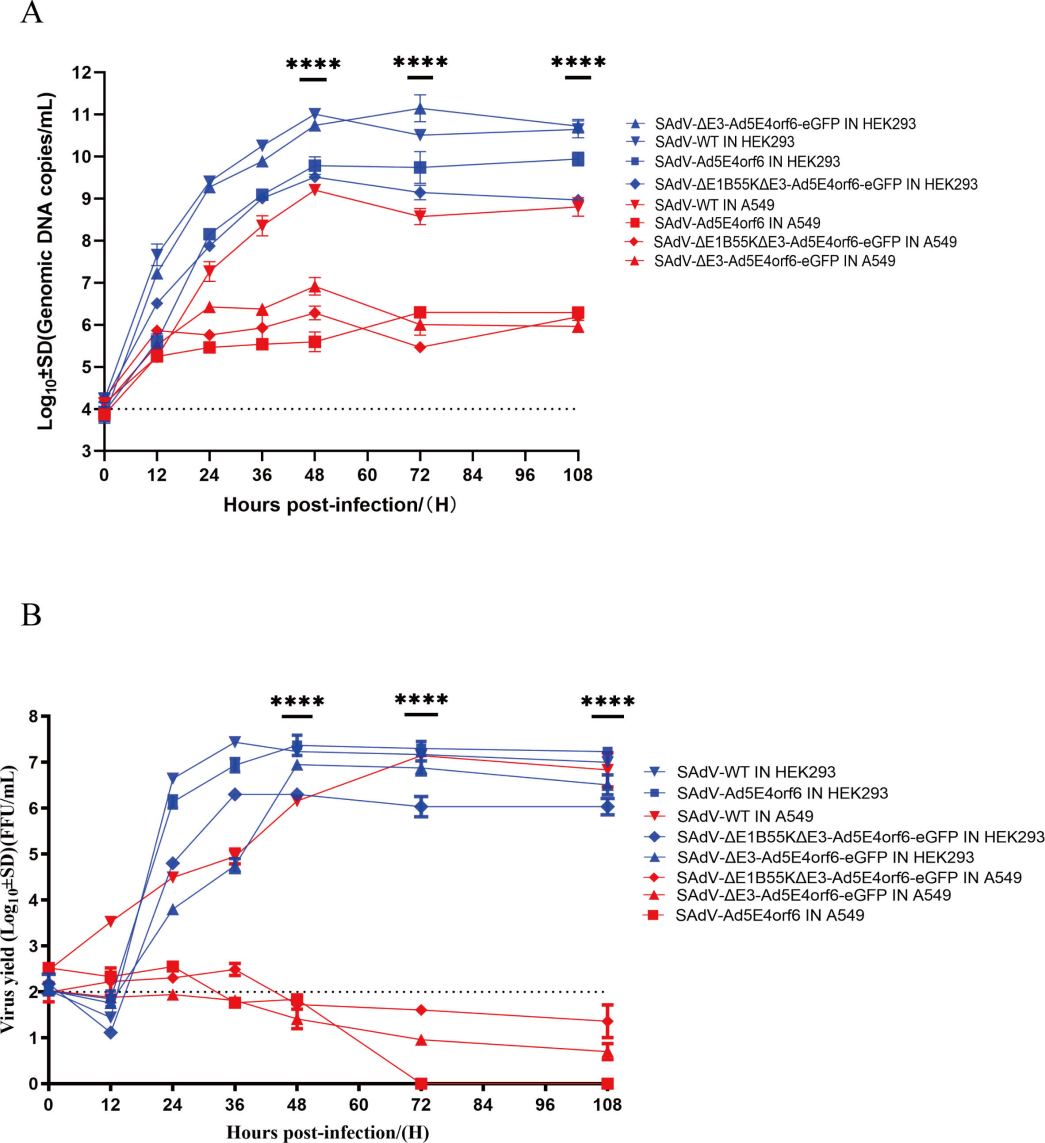
Viral growth kinetics of the constructs in HEK293 and A549 cells, respectively. One-step growth curves for constructs SAdV-Ad5E4orf6, SAdV-ΔE3-Ad5E4orf6-eGFP, and SAdV -ΔE3ΔE1B55K-Ad5E4orf6-eGFP were determined and compared to SAdV GZ3-12. Cells were inoculated at MOI (multiplicity of infection) of 0.5 and harvested at 0, 12, 24, 36, 48, 72, and 108 h. (**A**) Viral genomic DNA copy numbers in the harvested cultures were determined by quantitative PCR using a qPCR kit (Takara Biomedical Technology (Beijing) Co., Ltd.; Beijing, China), and (**B**) virus yield calculated using FFU (Fluorescent Forming Unit). The dashed line represents the detection limit of the method. Red lines represent viruses grown in HEK293 cells, and blue lines represent viruses grown in A549 cells.

The replication efficiencies of rescued viruses from the infectious clone and SAdV GZ3-12 were compared using qPCR quantification of the genomic DNA copy numbers and by direct immunofluorescence to compare viral loads (Fig. 9). Panel A shows the change in DNA copy numbers, and panel B presents the change of infectious virus titers at different time points. The red lines represent the viruses cultured in A549 cells, and the blue lines represent the viruses cultured in HEK293 cells. In general, in addition to the SAdV GZ3-12, the other three replication-deficient viruses maintained only low levels of replication in A549 cells due to the absence of HAdV-5 E1 genes. Among the three replication-deficient viruses, those with a deletion of the E3 region and substitution of E4orf6 had the level of viral DNA replication closest to that of the wild-type virus, whereas those with the deletion of the E1B55K gene had the lowest level, possibly because the human adenovirus E1B55K protein expressed within HEK293 cells was not at sufficient levels or did not fully function during replication of the simian adenovirus (Fig. 9A). As seen from the number of infectious viruses packaged, simian adenovirus could not produce progeny virus in A549 cells after the replacement of the E4orf6 gene, indicating that the E4orf6 gene plays an important role in the replication process of simian adenovirus. In HEK293 cells, the number of infectious viruses peaked and subsequently reached a plateau at 48 hpi. Of the three replication-deficient viruses, adenoviruses that had the E4orf6 gene replaced with Ad5E4orf6 gene produced the number of infectious viruses closest to that of the wild-type virus at the respective time points. Viruses with the E3 region deleted had slightly lower numbers of infectious viruses at each time point than SAdV GZ3-12. However, SAdV constructs with both the E1B55K gene and the E3 region deleted showed a 5- to 10-fold reduction in the number of infectious viruses at all time points, compared with SAdV GZ3-12 (Fig. 9B). This indicated that the genes encoded by E3 regions and E1B55K exert some influence during SAdV replication and proliferation.

### SAdV GZ3-12 infects cells expressing hDSG2 or hCAR

**Fig 10:**
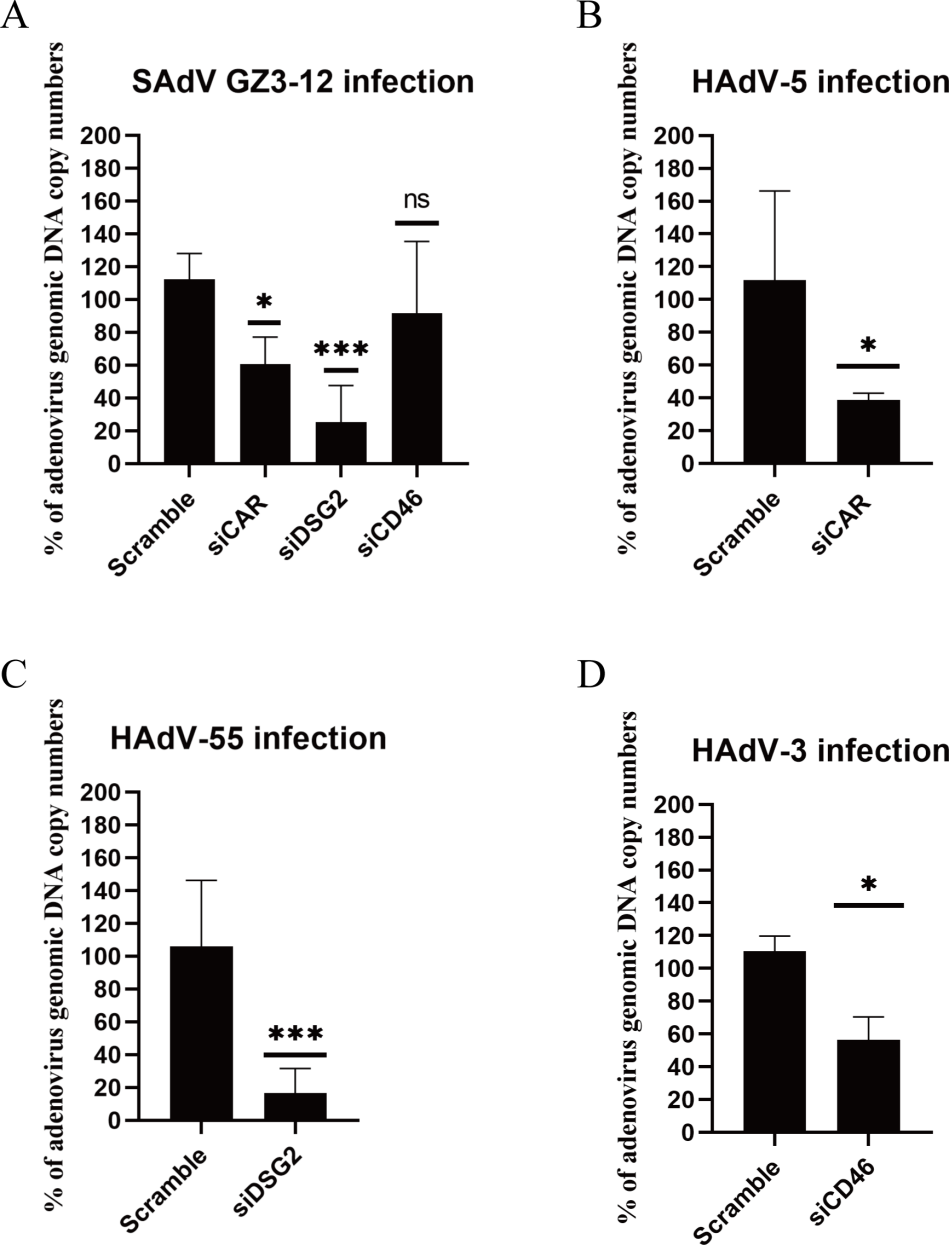
Knockdown of hDSG2 and hCAR reduces SAdV GZ3-12 infection. (**A**) Knockdown of hDSG2 and hCAR significantly reduced SAdV GZ3-12 genomic DNA copy numbers post-infection in A549 cells. A549 cells pre-transfected with siRNAs were infected with SAdV GZ3-12 at 1 MOI. At 48 hpi, the viral genomic DNA in cells was quantified. Percent infection was calculated as the percentage of the viral genomic DNA copy number in cells transfected with siRNAs versus that in cells transfected with the scrambled control. (**B**) Knockdown of hCAR reduced HAdV-5 genomic DNA copy numbers post-infection in A549 cells. (**C**) Knockdown of hDSG2 reduced HAdV-55 genomic DNA copy numbers post-infection in A549 cells. (**D**) Knockdown of hCD46 reduced HAdV-3 genomic DNA copy numbers post-infection in A549 cells.

To evaluate the receptor usage of SAdV GZ3-12, the expression of hDSG2, hCAR, or hCD46 in A549 cells was knocked down using RNA interference, respectively, as described previously (37). Knockdown of hDSG2 or hCAR individually significantly reduced GZ3-12 infection, whereas knockdown of hCD46 alone showed no significant reduction of GZ3-12 infection (Fig. 10A). For comparison, knockdown of hCAR, hDSG2, or hCD46 individually reduced HAdV-5, HAdV-55, and HAdV-3 infection significantly (Fig. 10B, C and D). These three types of adenoviruses have been confirmed to infect cells via recognizing the host CAR (67), DSG2 (37, 38), and CD46 (68) as receptors, respectively. This result indicates that GZ3-12 mainly infects cells via recognizing hDSG2 and hCAR receptors.

## DISCUSSION

Adenovirus vectors are widely used as gene therapy and vaccine vectors because of their potential for shuttling large heterologous gene(s), easy manipulation, and their inability to be integrated into the host genome (69). However, HAdV-5, the most widely used Ad-based vector, is a commonly circulated respiratory pathogen, particularly in children, and may elicit robust immune responses due to its high seroprevalence. It has been reported that the seropositive rate of HAdV-5 neutralizing antibodies in some populations is as high as 80% (21). To circumvent pre-existing nAbs, SAdVs and less commonly circulated HAdV genotypes have been developed for use as vectors (70 – 72). Still, potentially pre-existing immunity remains an important consideration and potential limitation in their applications as suitable vectors. Therefore, as reported in this study, a novel SAdV from macaques was identified, isolated, characterized, and explored as a vector candidate. Compared with HAdV-5, the seropositivity of nAb sera against SAdV GZ3-12 across several populations is about 10%. By replacing E4orf6 and deleting the E1B55K and E3 regions, the modified SAdV GZ3-12 construct may be a good vector candidate due to its presumed lack of immunogenicity and its capacity to carry heterologous genes.

In this study, 33.9% (39/115) of macaque feces samples tested positive for an SAdV by PCR screening. All of the fecal samples were collected from healthy, asymptomatic adult monkeys. This SAdV-positive rate is higher than the prevalence (17.9%) reported in the literature (73), which suggests that SAdVs in monkeys may be more common than previously thought. In this study, 39 putative SAdV-related sequences were obtained. Surprisingly, up to 51% (20/39) of the sequences are more similar to HAdVs than SAdVs, and 85% (17/20) of these have sequence similarities to genotypes of the HAdV species D clade (Fig. 1). Currently, more than 50 serotypes and genotypes of SAdVs have been isolated and characterized into nine SAdV species (SAdV A–I) (74, 75), a taxonomic distinction that has been recently discussed (2). The sequence relationships, we identified may be due in part to the close proximity of monkeys and apes in zoological parks and other captive primate colonies with human caregivers and even the general public, which may lead to adenovirus transmission, both zoonotic and anthroponotic. Such cross-transmission may also result in recombination, as reported in the literature (29, 30, 32, 33, 76) . An alternative scenario is that newly captured/introduced primates may harbor adenoviruses from the wild with sequence similarity to HAdV-D genotypes, and may pass these onto the colony.

The biggest difficulty in constructing infectious adenovirus clones is that the genome is large, approximately 28–44 kb (77), making direct ligation difficult. There are four current methods to construct recombinant adenoviruses. The first method is to ligate the DNA fragment of adenovirus genome directly to an endonuclease-treated fragment containing the inserted gene (78); this method has proven of limited utility because of the low efficiency of large fragment connection and the need for a unique restriction endonuclease site. The second method is through homologous recombination in mammalian cells. The replication-deficient virus produced by recombination needs to be replicated in a specific cell line (such as HEK293 cells), and the recombinant virus then needs to be screened through plaque purification (79, 80). Although this method can be useful, the efficiency of homologous recombination is low (81). Our team has previously used homologous recombination in BJ5183 competent bacteria to construct adenovirus vector vaccines (46). Compared to the method of homologous recombination in mammalian cells, the method we developed has higher efficiency. The plasmid can be used repeatedly over time. The third method is to amplify the adenovirus genome by PCR and then ligate it into the pUC19 plasmid. Although this method can delete unnecessary genes randomly and unexpectedly, the entire process is very time-consuming and cannot guarantee that mutations will not occur during the amplification process (81). In addition, the GC content of SAdV genomes in this study falls between 46.70% and 62.62%, with the GC contents of 19 out of all 29 genomes more than 55%. High GC content may reduce the success rate of PCR amplification of SAdV gene for homologous recombination. The fourth method is bacterial artificial chromosomes coupled to bacteriophage λ red recombination (recombineering, Red/ET recombination) technology (82, 83). Red/ET recombination uses λ phage-derived recombination proteins that mediate effective recombination of linear DNA fragments into the target sequences, requiring only very short (usually 35–50 bp) homologous sequences (84). However, in the latter case, degradation of linear DNA in *E. coli* must be prevented, and short-terminal repeats within the homology arms of the vector may lead to a substantial background due to a high frequency of vector circularization (85). Therefore, we chose the Gibson assembly method, which has the advantages of easy manipulation, repeatability, and no need for large overlapping sequences. Using this method in prior work, we successfully constructed an infectious clone of Ad14 (48).

As a readout of transfection efficiency, we replaced the E3 region of SAdV GZ3-12 with the eGFP gene in HEK293 cells transfected, aggregated fluorescent cells were observed, which indicated that the virus infected and replicated in the cells. However, fluorescence was only observed in a limited number of cells and eventually fluorescence disappeared over time without CPE (Fig. 6A and B). However, the virus lacking E3 region could be rescued in complementing HEK293-E3 cells and replicated and expressed GFP very well. It also caused typical CPE. This observation indicated that the E3 coding region may play an important role in the replication of simian adenoviruses, and that the deletion of this region may interfere with virus replication or assembly.

To circumvent the reduced replication that accompanied deletion of region E3, we replaced the E4orf6 gene with the HAdV5-E4orf6 gene. The complex formed by the E1B55K protein and E4orf6 protein binds to E2F, disrupts the E2F/Rb complex, and stimulates viral DNA replication and production of viral progeny (66, 86). Likely, the Ad5 E1B55K protein provided by HEK293 cells interacts with the HAdV E4orf6 protein to promote adenovirus replication in this human cell line. The E4orf6-replaced virus replicated successfully in HEK293 cells, similar to the wild-type virus, but not in A549 cells, which do not express the Ad5E1B55K protein (Fig. 9). SAdV-ΔE3-Ad5E4orf6-eGFP was also rescued successfully in HEK293 cells. This result indicates that the Ad5E4orf6 protein interacts with the E1B55K protein of HEK293 cells, and compensates for the deletion of the E3 region. SAdV-ΔE3ΔE1B55K-Ad5E4orf6-eGFP contains a deletion of the E1B55K gene, which could replicate and package only in HEK293 cells, and also allows an increase of the capacity for heterologous genes. Finally, through siRNA interference experiments, we confirmed that GZ3-12 can recognize both CAR and DSG2 receptors, since hCAR and hDSG2 receptors are abundantly expressed in human cells (87, 88), GZ3-12 has extensive infectivity to human cells.

This report presents the construction and characterization of a candidate vector for human gene therapy and vaccine development. We used a novel SAdV isolated from captive macaques, to minimize potential pre-existing human immune responses. During the collection and characterization, we observed that SAdVs were widely present in captive macaques, and could be detected and isolated in a non-invasive manner. Using one such isolated virus, GZ3-12, a replication-deficient vector was constructed. In a small survey, neutralizing antibody titers against this SAdV in the general population were low, suggesting that its use could largely avoid pre-existing immune responses. Our construct was able to infect several different human cancer cell lines and express heterologous proteins. During the course of developing this vector, it was also observed that constructs lacking an E3 region could not produce infectious progeny in human cell lines. In constructs with a deleted E3 region, however, viruses could be induced to produce infectious progeny by the substitution of the native E4orf6 with the homologous E4orf6 from HAdV-5 as long as HAdV E1B55K was also present. Given this finding, we showed that the E3 region of the macaque adenovirus genome plays a critical but as yet undefined role in its replication in human cells, but the absence of this region could be compensated for by the E4orf6 from HAdV-5 and the E1 expression intrinsic to human HEK293 cells.

## Data Availability

The annotated GZ3-12 genome was submitted to GenBank (accession number OP921949).

## SUPPLEMENTAL MATERIAL

Fig. S1， Fig. S2，Table S1 jvi.01014-23-s0001.docx Technical roadmap, reverse transcription PCR and primer list.

ASM does not own the copyrights to Supplemental Material that may be linked to, or accessed through, an article. The authors have granted ASM a non-exclusive, world-wide license to publish the Supplemental Material files. Please contact the corresponding author directly for reuse.
