# Supplementary material for: Isolation of novel simian adenoviruses from macaques for development of a vector for human gene therapy and vaccines
Source: J Virol. 2023 Sep 15;97(10):e01014-23. doi: 10.1128/jvi.01014-23 (PMC10617444; doi:10.1128/jvi.01014-23)
Supplement: Fig. S1， Fig. S2，Table S1 — Technical roadmap, reverse transcription PCR and primer list. [file jvi.01014-23-s0001.docx]

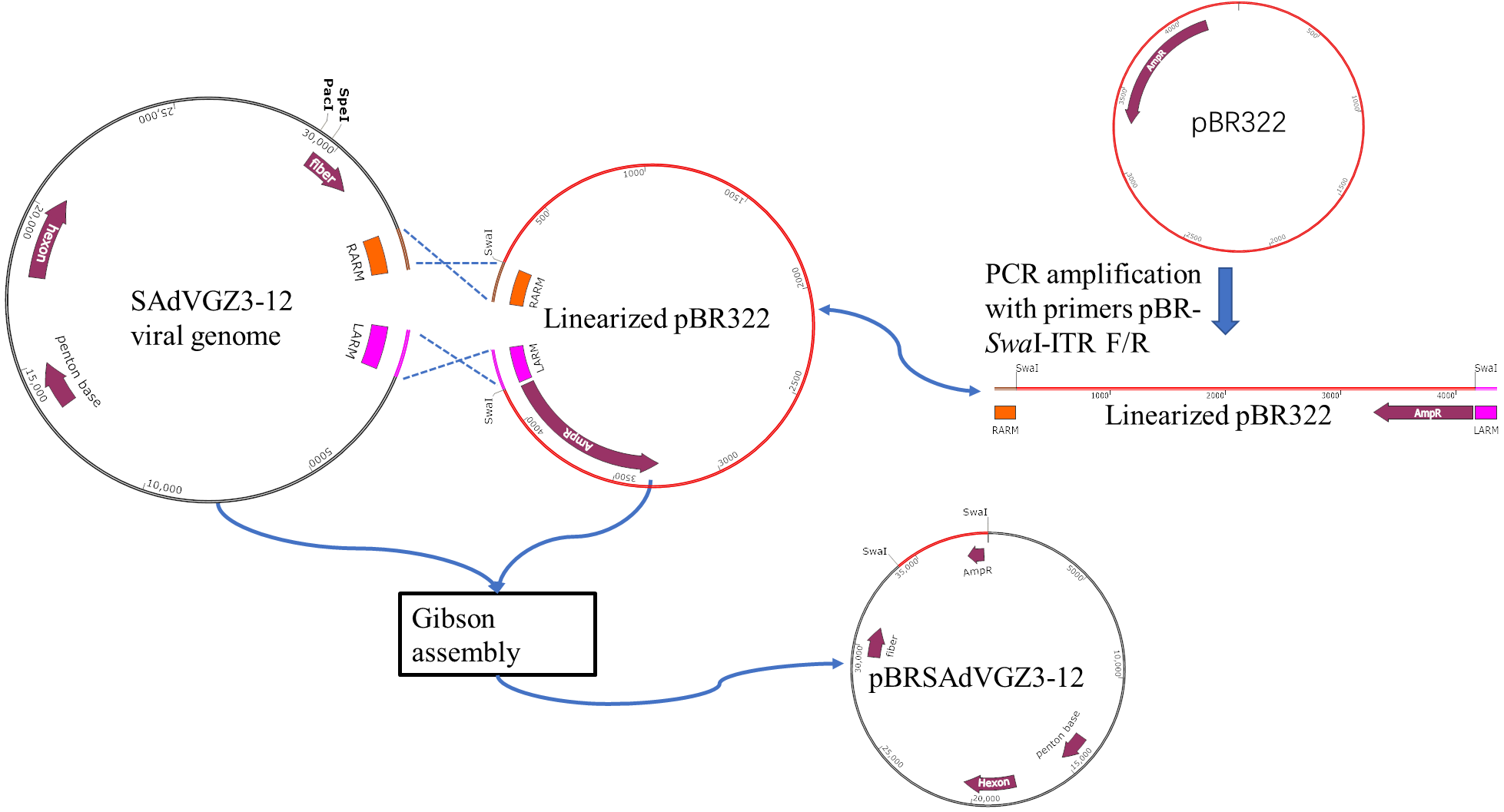


A


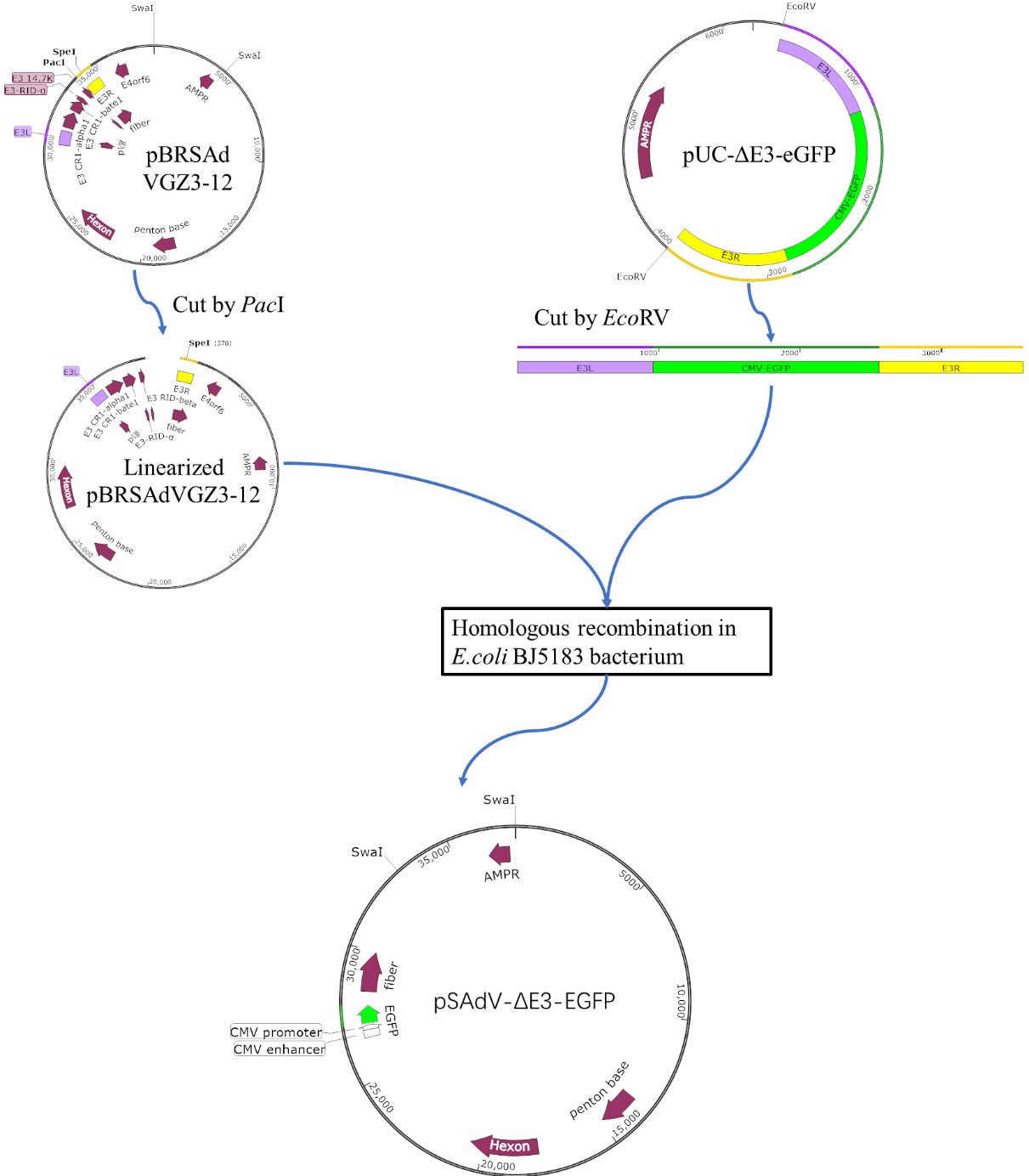


B


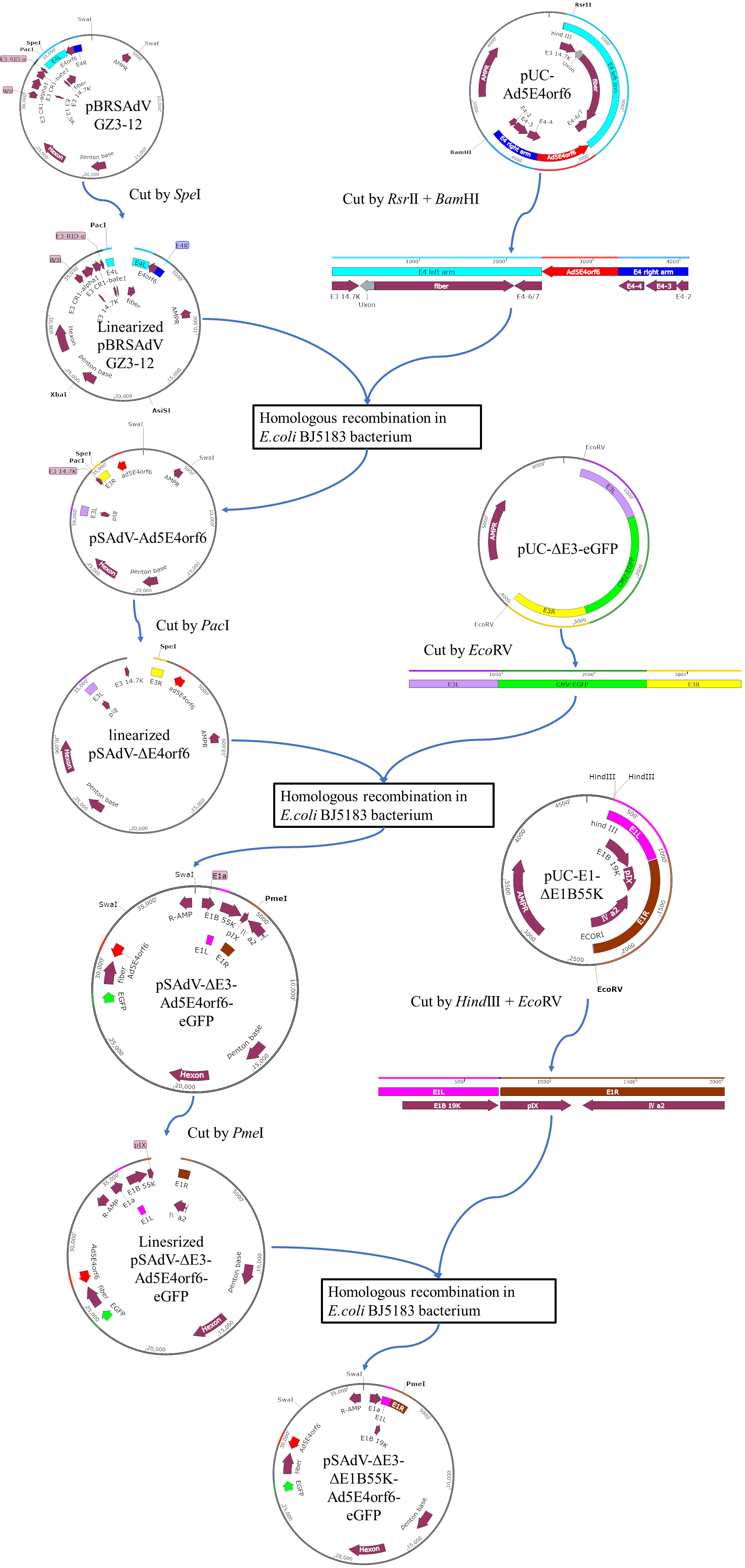


C

**Figure S1.** Technical roadmap for constructing SAdV plasmid vectors. A, Construction of plasmid containing the entire full-length genome of GZ3-12; B, Deletion of the E3 region of pBRSAdVGZ3-12 and insertion of eGFP gene; C, Replacement of SAdV E4orf6 with HAdV-5 E4orf6, and deletion of the E3 region and E1B55K gene of SAdV GZ3-12.


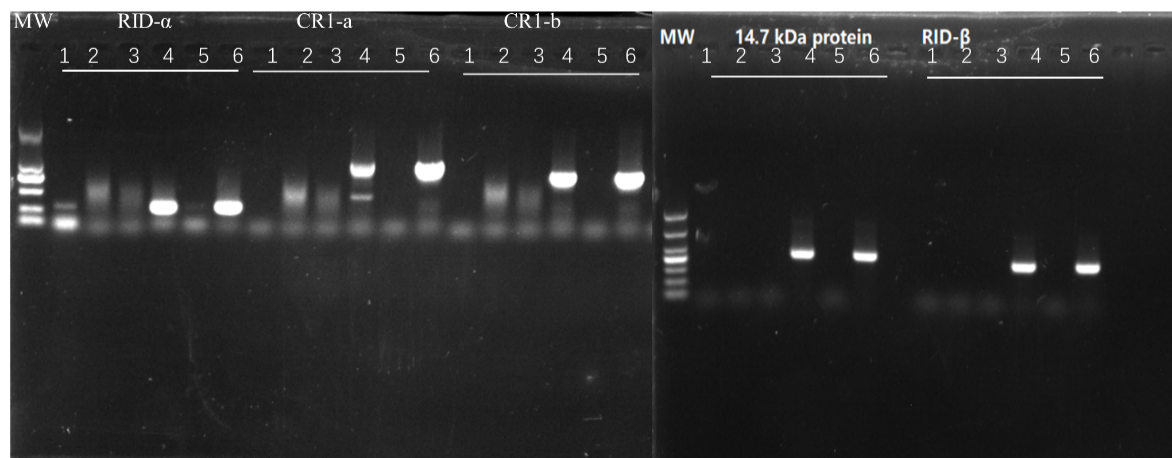


**Figure S2.** **Detection of E3 genes in HEK293-E3 cell line by reverse transcription PCR.** The size of each gene is as follows: CR1-α, 1.1 kb, CR1-β, 777 bp, RID-α, 276 bp, RID-β, 315 bp, 14.7K, 378 bp. Lane 1: Negetive control. Lane 2 and 3: DNase-treated RNA from HEK293-E3 cells and HEK293-empty cells. Lane 4 and 5: cDNA from HEK293-E3 cells and HEK293-empty cells. Lane 6: Positive control.

**Table S1. Primers used in this study.** PCR primers targeting conserved sequences in select adenoviral genes were designed for several purposes, as noted in the table. Each primer pair consists of a forward (F) and a reverse (R) primer. Predicted PCR amplicon sizes are noted.

| **Primer** | **Sequence (5’-3’)** | **PCR product(bp)** | **Purpose** |
| --- | --- | --- | --- |
| Hexon F | GCCCCARTGGGCRTACATGCACATC | 300 | Detection of adenovirus in fecal samples |
| Hexon R | GCACSCCSCGRATGTCAAAG |  |  |
| pBR-SwaI-ITR F | ***GTTAACTCGGTCGCCATCTTGCGGTGTTATATTGATGATG****atttaaat*ctcatgtttgacagcttatcatcg* | 4453 | Amplification of the pBR322 vector and the addition of 5’ ITR homologous arm (40 bp), *Swa*Ⅰ restriction sites at both ends |
| pBR-SwaI-ITR R | ***GTTAACTCGGTCGCCATCTTGCGGTGTTATATTGATGATG****atttaaat*cttgaagacgaaagggcctcg |  |  |
| SAdV-L-F | TCAAACATCTCGGCTTACC | 1429 | Screening of positive infectious clones (targeting the left ligation region) |
| SAdV-L-R | TCCCTTATGCGACTCCTG |  |  |
| SAdV-R-F | TTCGCCAGTTAATAGTTTG | 1383 | Screening of positive infectious clones (targeting the right ligation region) |
| SAdV-R-R | GAGGATCGTCTCCCAGAG |  |  |
| E4LF | ***ACCATGATTACGCC****AAGCTT*ATCTGTCTGCAGCTACTTTCATC | 2502 | Amplification of upstream sequences of E4orf6 |
| E4LR | **ATGACTCTACCCCCATGTAG***CCACGGTGGACATGGAGGAG* |  |  |
| Ad5 E4orf6 F | ctacatgggggtagagtcat | 905 | Amplification of HAdV-5 E4orf6 |
| Ad5 E4orf6 R | atgactacgtccggcgttcca |  |  |
| E4RF | **TGGAACGCCGGACGTAGTCA**TAGCATACAAGTTGGGACCTA | 803 | Amplification of downstream sequences of E4orf6 |
| E4RR | **GGTACCCGG***GGATCC***TCTAG**AGCTAATTGTTGCTCTGATTT |  |  |
| E3LF | **AAAACGACGGCCAGTGAATT***CGATATCGATTCCCACGCCTTAC* | 1145 | Amplification of upstream sequences of E3 region |
| E3LR | **ATTGATTACTATTAATAACT*TCGCCGTAGTACCAAGT*** |  |  |
| eGFP F | AGTTATTAATAGTAATCAATTAC | 1604 | Amplification of CMV promoter and eGFP |
| eGFP R | TGCAGTGAAAAAAATGC |  |  |
| E3RF | **GCATTTTTTTCACTGCACCA**AGGGAACCATAA | 1067 | Amplification of downstream sequences of E3 region |
| E3RR | **GACCATGATTACGCCAAGCTT***GATATC*CATAGCCCGTCTGAAC |  |  |
| pLenti-E3F | **AGACACCGACTCTAGATATC**ATGACTGATGTCGAGCCCGC | 3233 | Amplification of the E3 region of SAdV GZ3-12 |
| pLenti-E3R | **TGGTCTTTGTAGTCAGCCCG**GGATCCTTAATTAAAGGGGATAGAAT |  |  |
| E1B55K-L-F | **GATTACGCCAAGCTTct***aagctt*ccgggtgactca | 737 | Amplification of upstream sequences of E1B55K |
| E1B55K-L-R | **CATGGTACCTGAAAA**CTACTCCTCCGCTGGAGGGT |  |  |
| E1B55K-R-F | **TTTTCAGGTACCATGA**GCGGATCAAGCAGCC | 1314 | Amplification of downstream sequences of E1B55K |
| E1B55K-R-R | **aaaacgacggccagt***gatatc*TTGCGCAACCTGCTTTCCAC |  |  |
| siCAR sence | GCCAGAAGUUUGAGUAUCATT | / | Knockdown of the expression of CAR receptor in cells |
| siCAR antisence | UGAUACUCAAACUUCUGGCTT |  |  |
| siDSG2 sence | CCAAUUGCCAAGAUACAUUTT | / | Knockdown of the expression of DSG2 receptor in cells |
| siDSG2 antisence | AAUGUAUCUUGGCAAUUGGTT |  |  |
| siCD46 sence | GCCUGUUAUAGAGAAACAUTT | / | Knockdown of the expression of CD46 receptor in cells |
| siCD46 antisence | AUGUUUCUCUAUAACAGGCTT |  |  |
| Scramble sence | UUCUCCGAAGGUGUCACGUTT | / | Negative control |
| Scramble antisence | ACGUGACACGUUCGGAGAATT |  |  |

*: The italicized and bold sequence is the ITR sequence of SAdV; the bold and underlined sequence is the overlapping sequence for constructing the Shuttle plasmid；the italicized and underlined sequence is the restriction endonuclease site introduced in the primer.
